# Stress experience and coping strategies in medical studies – insights and a discussion of preventive measures

**DOI:** 10.3205/zma001730

**Published:** 2025-02-17

**Authors:** Iris Warnken, Sabine Polujanski, Thomas Rotthoff, Ann-Kathrin Schindler

**Affiliations:** 1 University of Augsburg, Faculty of Medicine, Medical Didactics and Education Research, DEMEDA, Augsburg, Germany

**Keywords:** coping sills, student, medical, perfectionism, retrospective studies, fear, burnout psychological, qualitative research

## Abstract

**Objective:**

A high level of stress and critical burnout values (27–56%) has been identified among medical students in numerous international research and review studies. The aim of this interview study was to gain insights into students’ perspectives on stressors, stress amplifiers and reactions, as well as the coping strategies they applied. The results will be used to discuss preventative measures in higher education.

**Methods:**

A total of 22 semi-standardised, semi-narrative interviews were conducted with medical students, students in their practical year and junior doctors to gain retrospective perspectives on their studies. All data were audio-recorded, pseudonymised, fully transcribed as well as structured and analysed using qualitative content analysis, based on Kaluza’s stress model.

**Results:**

Study-related causes (e.g. the amount of material), private issues (e.g. social conflicts) and aspects arising during clinical work phases (e.g. complexity of tasks) were named as stressors. Individual stress amplifiers, such as perfectionism, were also described. The respondents showed stress reactions, such as doubts and fears. The coping strategies described were varied, but some were seen to be effective only in the short term.

**Conclusion:**

The ability to cope with stress must be consciously learned and reflected upon across various causative areas. In particular, the discussion of mental strategies for dealing with repeatedly described stress amplifiers, such as one’s own perfectionism, appears to be a behavioural prevention measure that is still little used by medical students. In terms of behavioural prevention, discourses on large amounts of learning material, increased support in the transition phase at the start of a degree course and more flexible studying for medical students (e.g. with a family) must be further developed.

## Zusammenfassung

**Zielsetzung:**

Für Medizinstudierende wurde ein hohes Stresserleben sowie kritische Burnout-Werte (27–56% der Medizinstudierenden) in zahlreichen internationalen Forschungs- und Übersichtsarbeiten festgestellt. Ziel dieser Interviewstudie ist es, Einblicke in eine studentische Perspektive auf Stressoren, Stressverstärker und -reaktionen sowie deren bereits eingesetzte Bewältigungsstrategien zu gewinnen. Entlang der Ergebnisse sollen Präventionsmaßnahmen im Studium diskutiert werden.

**Methodik:**

Insgesamt wurden 22 teilstandardisierte, teilnarrative Interviews mit Medizinstudierenden sowie Studierenden im Praktischen Jahr und Assistenzärzt*innen für eine retrospektive Perspektive auf das Studium durchgeführt. Alle Daten wurden als Audio aufgezeichnet, pseudonymisiert, vollständig transkribiert und mittels qualitativer Inhaltsanalyse inhaltlich entlang des Stressmodells nach Kaluza strukturiert und analysiert.

**Ergebnisse:**

Als Stressoren wurden studiumsbezogene (z. B. Stoffmenge), private (z. B. soziale Konflikte) und im Rahmen klinischer Arbeitsphasen sich ergebende Aspekte (z. B. Komplexität von Aufgaben) genannt. Ebenfalls wurden individuelle Stressverstärker wie z. B. Perfektionismus beschrieben. Als Stressreaktionen zeigten die Befragten z. B. Zweifel und Ängste. Die beschriebenen Bewältigungsstrategien sind vielfältig, werden aber teilweise als nur kurzfristig wirksam reflektiert.

**Schlussfolgerung:**

Die Kompetenz für Stressbewältigung muss für variierende, verursachende Bereiche bewusst erlernt und reflektiert werden. Vor allem die Thematisierung mentaler Strategien zur Bearbeitung wiederholt beschriebener Stressverstärker wie des eigenen Perfektionismus erscheint als eine für Medizinstudierende noch wenig genutzte verhaltenspräventive Maßnahme. Verhältnispräventiv muss der Diskurs über hohe Stoffmengen, verstärkte Begleitung in der Transitionsphase zu Beginn des Studiums oder flexibleres Studieren von Medizinstudierenden, bspw. mit Familie, weiterentwickelt werden.

## 1. Introduction

**Figure 1:**
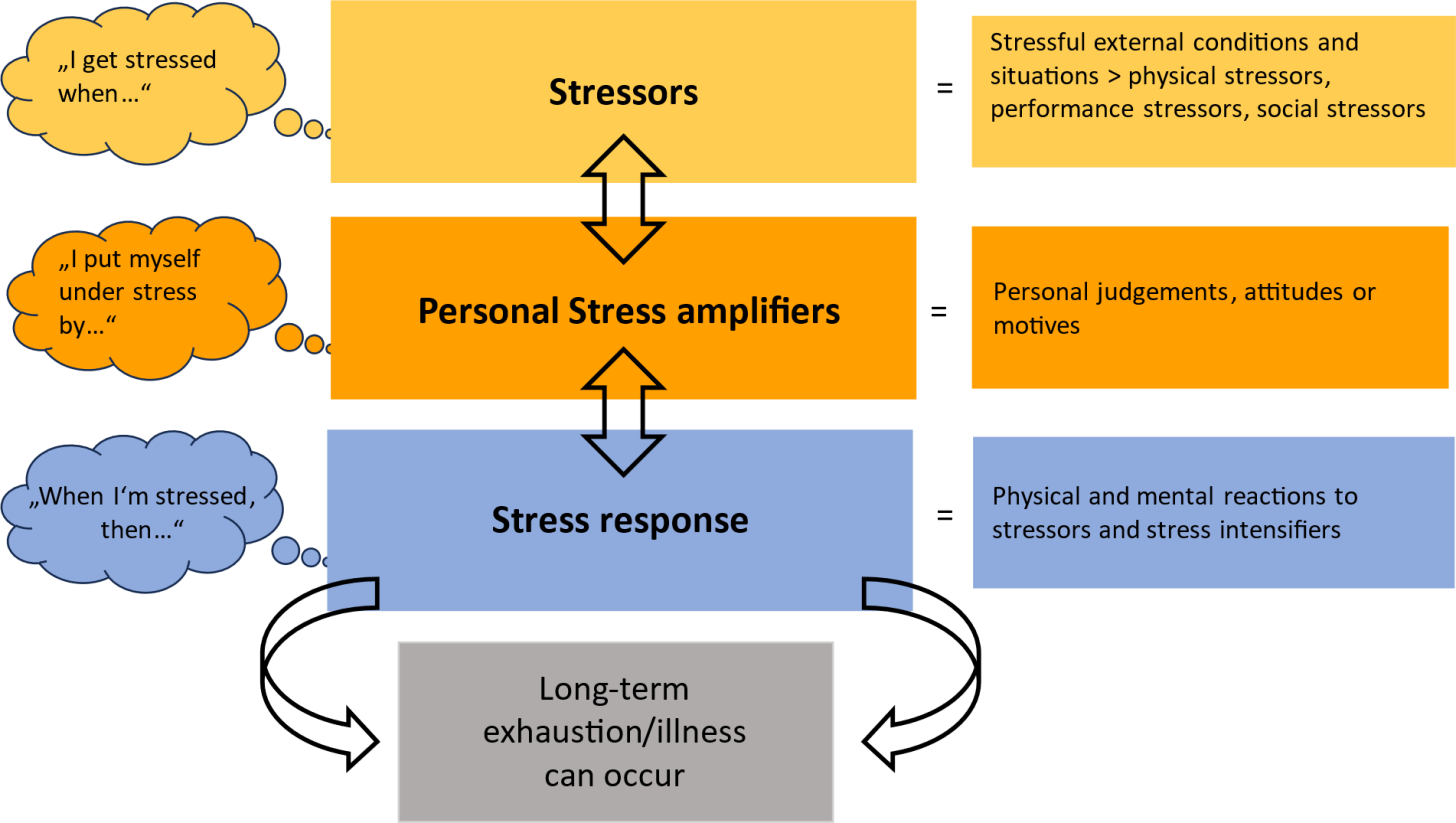
The levels of stress, according to Kaluza [11]

Stress is understood as a negatively experienced consequence of a discrepancy between the impact of pressures and individually perceived coping strategies influenced by previous experience [1]. Across disciplines, it is recognised that students sometimes experience a high level of stress that can lead to exhaustion (as a facet of burnout) without suitable coping strategies [2], [3]. For medical students, critical burnout values (27–56% of medical students) have been identified in numerous international research and review studies [4], [5], [6], [7], [8]. A better understanding of the stress experiences of medical students is considered relevant for the early prevention of burnout among doctors [4], [9]. According to Kaluza (see figure 1 (Fig. 1)), external stressors, such as high study requirements, or individual stress intensifiers, such as a perfectionist attitude, are responsible for perceived stress [10]. In response, physical and psychological stress reactions can follow, which can lead to exhaustion and illness [10]. The coping strategies used include not only active and successful coping but also enduring, tolerating or denying – that is, any form of endeavour to deal with stressors [11]. 

While short-term stress can have a stimulating effect, long-term stress – when an organism’s ability to adapt is overtaxed – is said to have a detrimental effect on health [12]. Susceptibility to stress and the use of coping strategies vary from person to person. Resilience to stress can and must be learned, consciously strived for and developed to avoid serious stress reactions [13].

This interview study builds on existing findings on the reasons why medical students experience stress. Among other things, an academic workload that is perceived as too high [14], [15], a good performance expected of oneself [14], [16], stressful experiences in the clinic [13] and private challenges [14], [17] have been identified. The aim is to use the strengths of qualitative research to provide further insights into individual experiences of stress and the coping strategies used during medical studies. Based on the results, we discuss behavioural and relational preventive measures. Behavioural prevention includes measures that address the personal behavioural changes of medical students, while relational prevention describes measures that can be implemented through faculty and curriculum development.

## 2. Methodology

### 2.1. Recruitment strategy

The call for participation in the guideline-based, semi-narrative interview study (December 2021 to March 2022) “Stress Experience in Studies and/or the Clinic” was sent by email to all students enrolled at the time of the survey in a new model degree programme in medicine at the University of Augsburg, which was implemented in the winter semester 2019/2020. All interested students (n=18) were included in the study and interviewed by two researchers (a psychologist with a Master’s degree and a sociologist with PhD who is experienced in conducting interviews). Due to the new degree programme, only students up to semester 5 were enrolled in the Augsburg model degree programme at the time of the study. To also include the experiences from higher semesters, two students in their practical year and two junior doctors were provided with retrospective views on the degree programme via private and collegial contacts. These conversations were conducted via a video call due to proximity and were recorded using an audio recorder.

### 2.2. Data collection

#### 2.2.1. Sample description

Between December 2021 and March 2022, 22 guided semi-narrative interviews (duration: *M*=66 minutes, min=39 min; max=102 min) were conducted by two interviewers, with the sample characterised in table 1 (Tab. 1). All participants were informed about the objectives of the study and the data protection conditions, and written consent to participate was obtained. The ethics committee of the Ludwig Maximilian University of Munich certified that the study was harmless (application 21-0711).

To maintain anonymity in line with data protection recommendations in the context of the still small Augsburg model degree programme, only the variables age, gender, semester and professional experience could be recorded to describe the sample. The sample consisted predominantly of female students. The majority of students already had professional experience in the healthcare sector, which was reflected in the maximum age values.

#### 2.2.2. Interview guide

The content of the interview guide (see attachment 1 ) was structured according to Kaluza’s stress model and, in terms of the subsequent analysis, according to Kuckartz’s qualitative content analysis method [18] and the principles of grounded theory, according to Corbin and Strauss [19]. The aim was to detect the greatest possible variability of information, opinions and personal experiences [19]. The interviewees were asked deductively derived key questions about stressors, their individually experienced stress reactions and their coping strategies. The question about stressors was deliberately open-ended and, initially, did not explicitly differentiate between “external stressors” and “individual stress intensifiers”. The interviewees’ independent naming of stressors in both categories was seen as a possible gain in knowledge from the interview study.

The research process changed iteratively according to grounded theory [19] between data collection, analysis and evaluation and served to further develop the guidelines. As a result, new aspects (e.g. sexism) emerged in the course of the surveys that were specifically queried in the subsequent interviews. Theoretical saturation with recurring elements signalled the end of the survey process. In terms of quality assurance, this process was continuously reflected upon and documented by means of written memos.

#### 2.2.3. Data analysis

The interviews were transcribed verbatim by an external provider in compliance with the GDPR criteria. The transcription was then validated by the first author, who is experienced in qualitative data analysis. The transcribed and pseudonymised interviews were analysed using MAXQDA 2022 Analytics Pro software (release 22.1.1.) in a step-by-step spiral coding process.

The first deductively derived main category scheme was based on the logic of the interview guidelines along the Kaluza model, with the categories being the stressors, stress reactions and coping strategies of the (former) medical students. The following further differentiating main categories were then inductively identified from the open question on stressors posed in the interview: stressors during studies, private stressors, stressors in clinical work and stress intensifiers. Further subcategories were then derived from the data material for all main categories.

**Figure 2:**
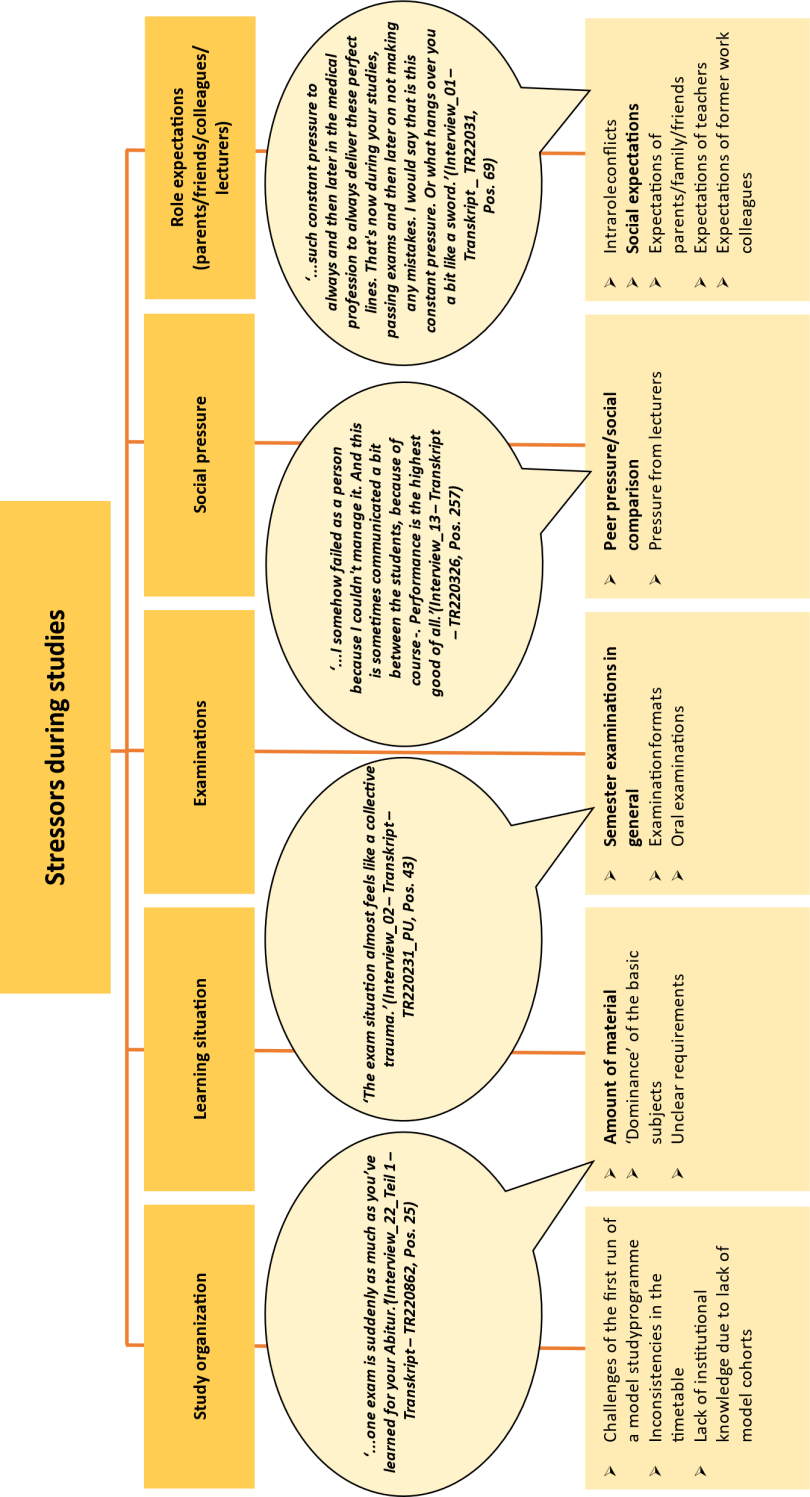
Stressors during studies

**Figure 3:**
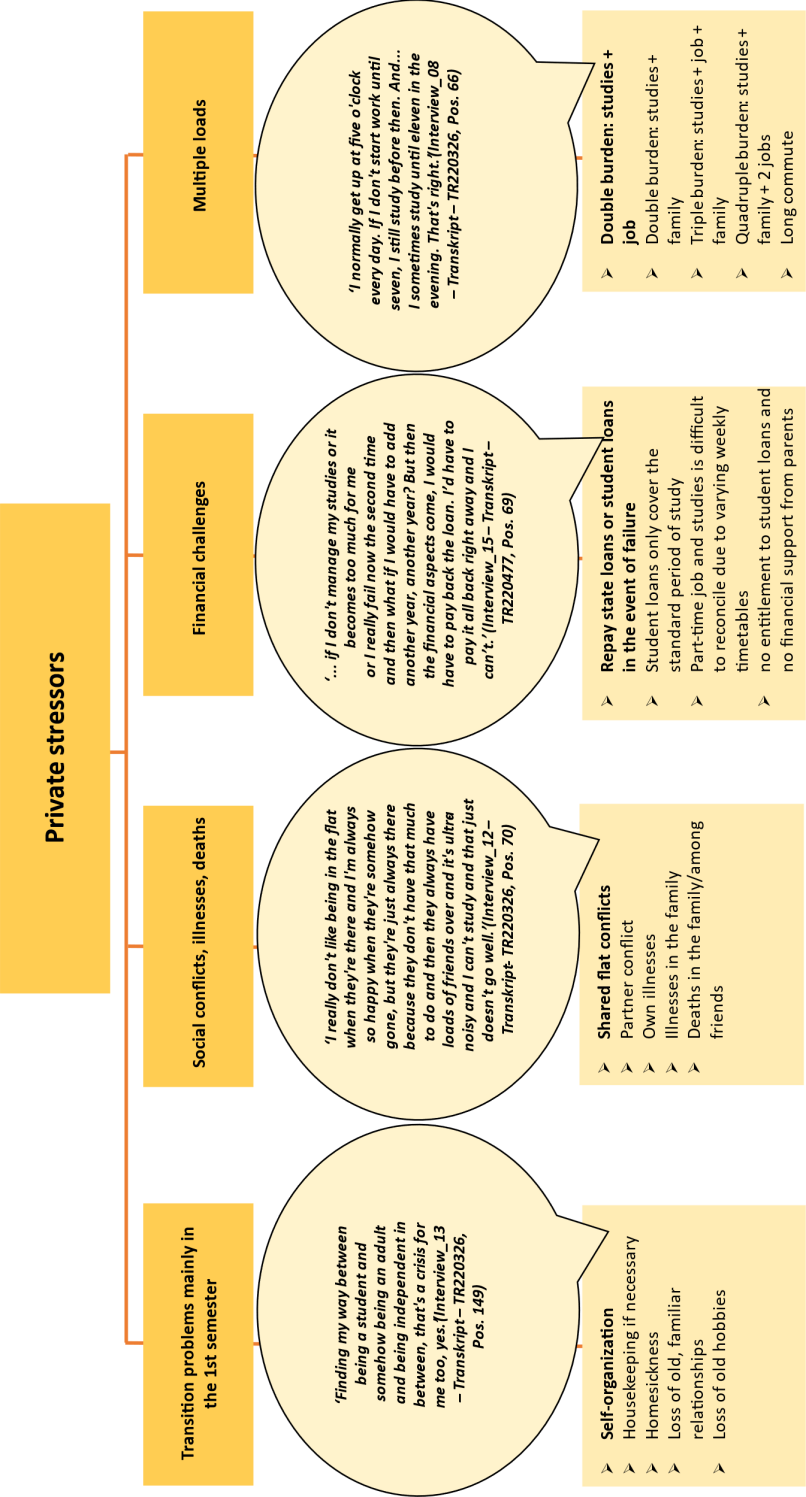
Private stressors

**Figure 4:**
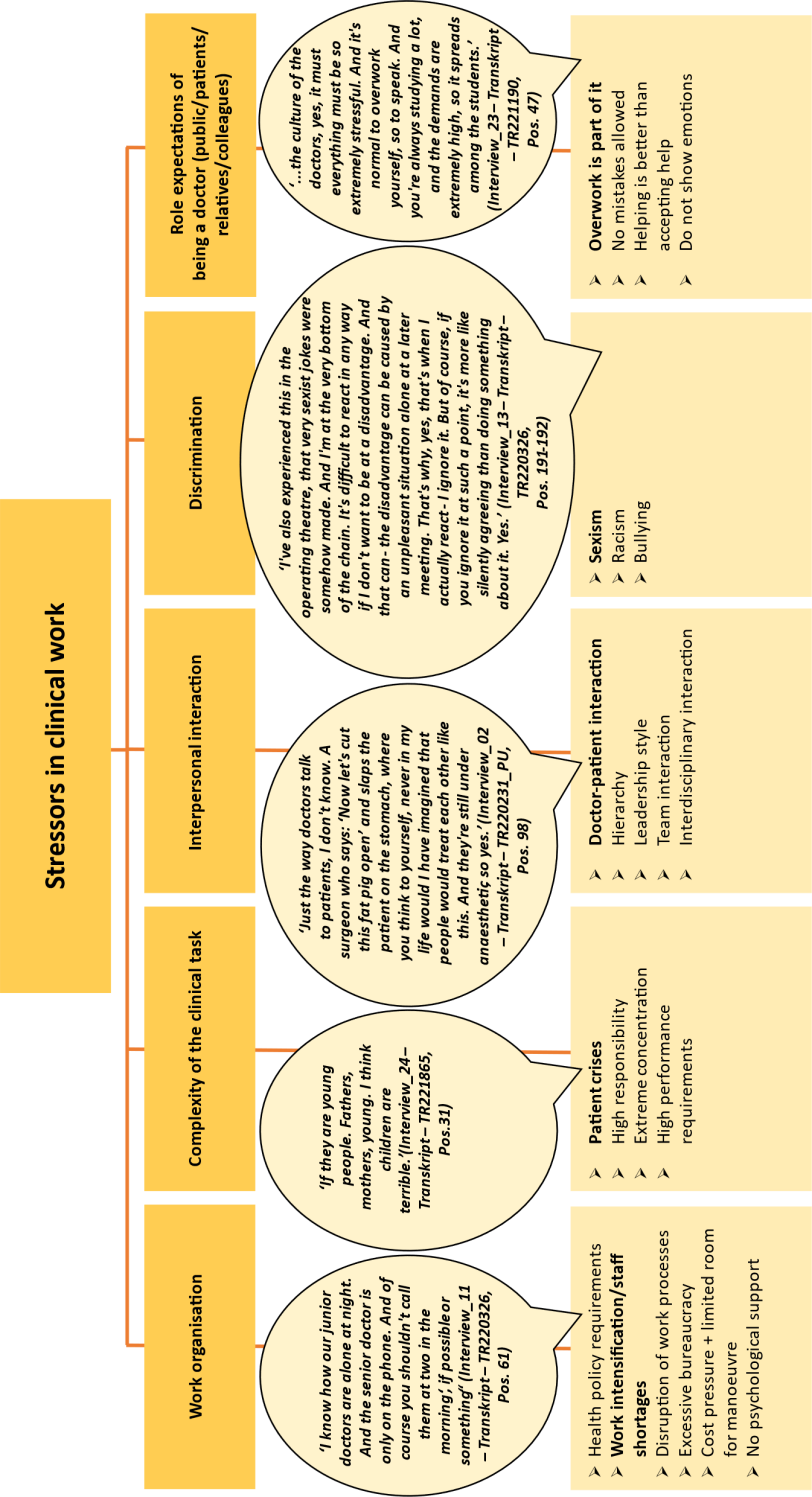
Stressors in clinical work

**Figure 5:**
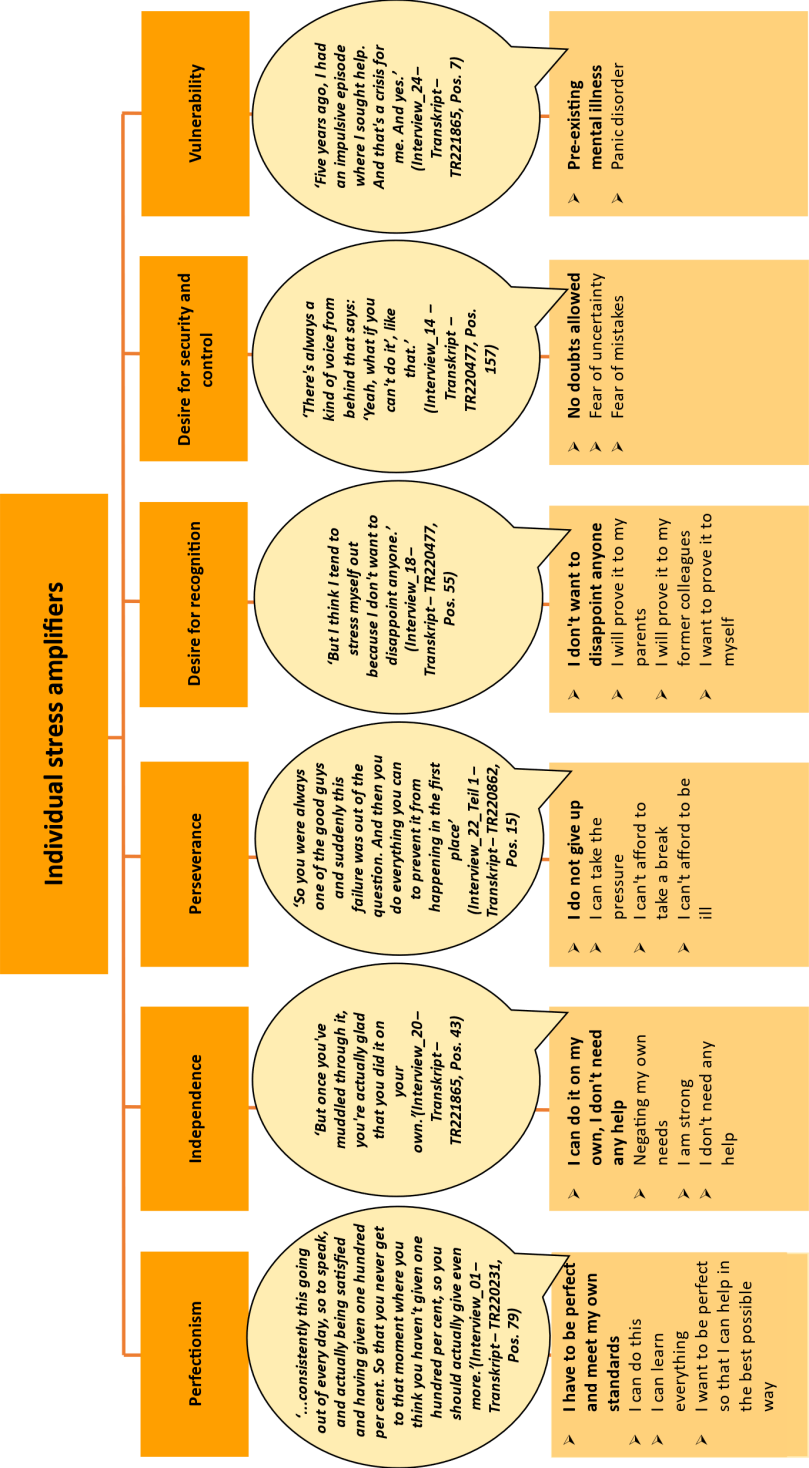
Individual stress amplifiers (cf. the five stress amplifiers, according to Kaluza [11])

**Figure 6:**
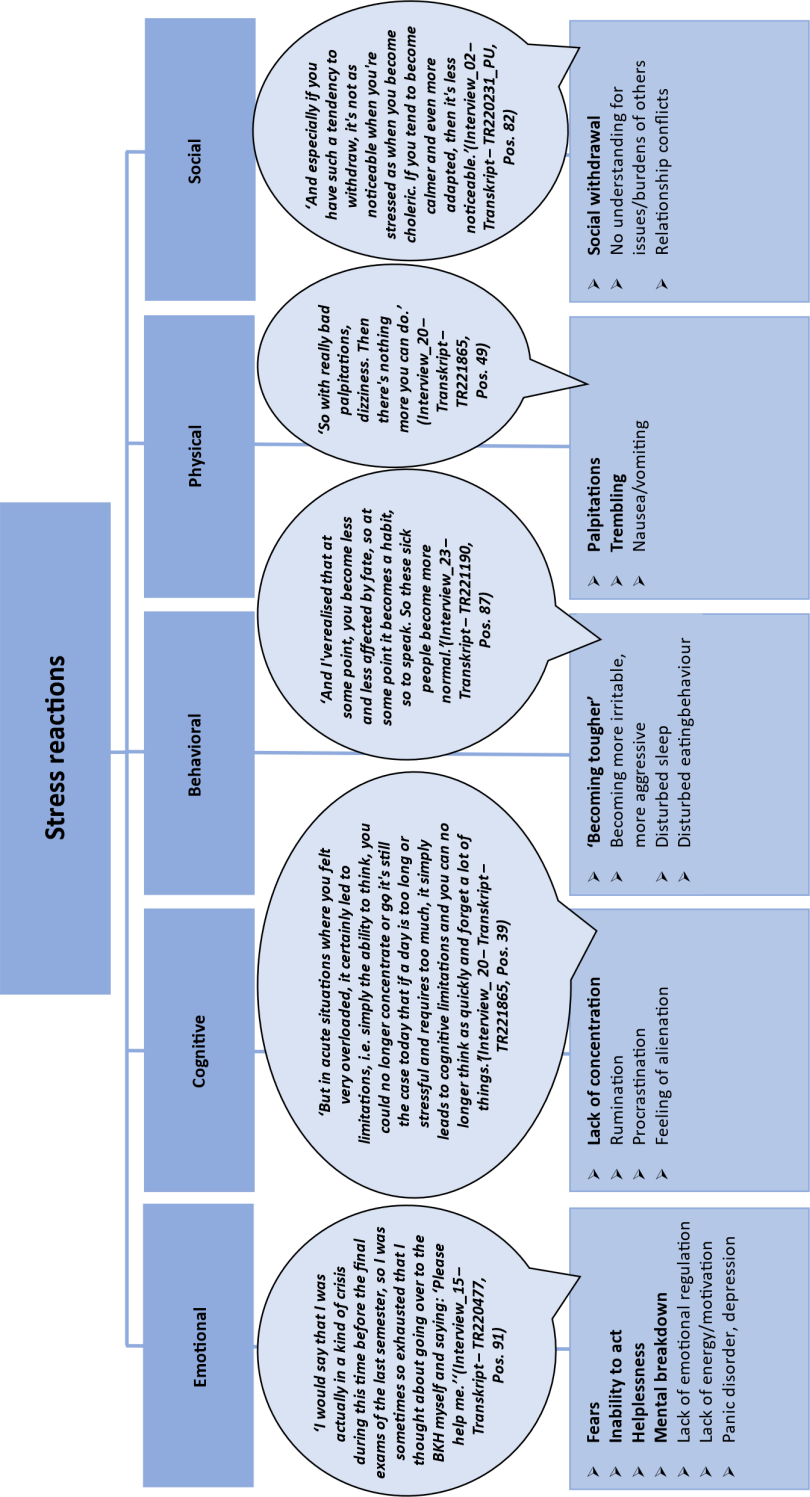
Stress reactions

**Figure 7:**
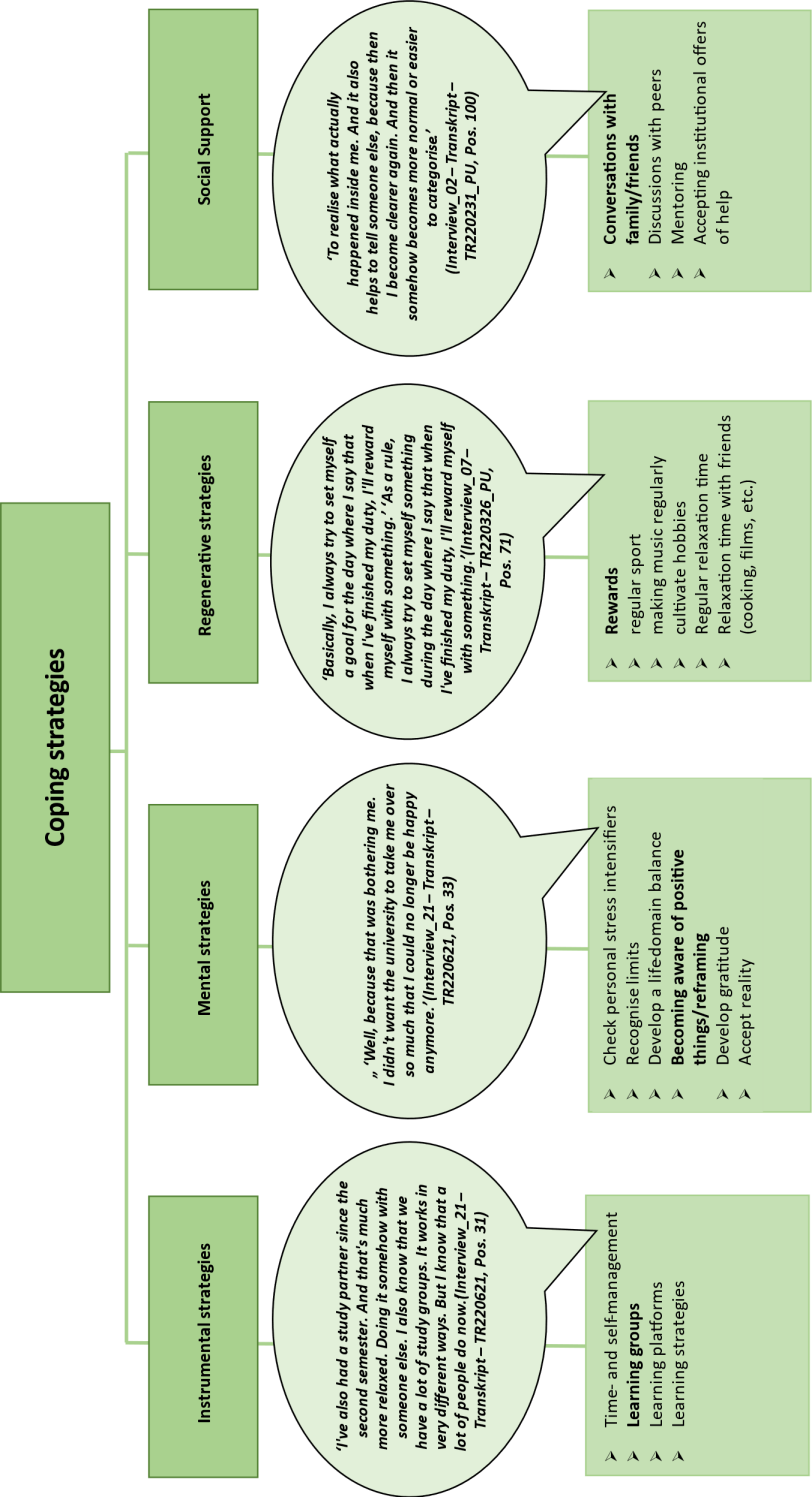
Coping strategies

The category system based on the first author’s initial coding was checked for plausibility by two second coders, and 25% of the data material was independently double-coded. Both second coders came from the superordinate project context and were familiar with the literature on mental health in medical students. In an iterative process, the category system was modified and discrepancies between the three coders were discussed until a consensus was reached. As the focus was on optimising the category system based on the data, no intercoder reliability was determined [20]. All categories thus determined by consensus were clearly defined and are listed in the results in figure 2 (Fig. 2), figure 3 (Fig. 3), figure 4 (Fig. 4), figure 5 (Fig. 5), figure 6 (Fig. 6) and figure 7 (Fig. 7) with exemplary quotations.

## 3. Results

### 3.1. Stressors

#### 3.1.1. Stressors during studies

The stressors mentioned were *organisation of studies, learning situation, examinations, social pressure* and the* role expectations* of medical students (see figure 2 (Fig. 2)).

In addition to the stressors perceived by some respondents and identified as challenges in a newly established degree programme, all respondents named the amount of material to be learned as the most significant stressor. Furthermore, unclear basic subjects and subjects perceived to determine time and content were described as stressful.

Oral examinations, new and unfamiliar examination formats (esp. OSCE) and relevant qualification milestones (e.g. module examinations or the first part of medical examinations) were described as stress-inducing. These stressors were supplemented by perceived pressure to perform and peer pressure (e.g. through social comparisons) as well as the communicated *role expectations* of parents, family, friends and former colleagues as well as social expectations.

#### 3.1.2. Private stressors

*Transition problems*, especially in the first semester, *social conflicts, financial challenges* and multiple loads were mentioned here (see figure 3 (Fig. 3)).

Around half of the respondents stated that they were burdened by *transition problems* in the first semester, such as homesickness, missing familiar relationships and hobbies and the challenge of having one’s own household for the first time. *Social conflicts* (personal), *illnesses and deaths* in the family or circle of friends, were also mentioned.

*Financial challenges* were discussed by individual interviewees. The interviewed students with career advancement grants, state loans or scholarships were also concerned about their grade point average or passing grade.

*Multiple loads*, such as due to the pursuit of secondary occupations – mostly in a trained profession in the healthcare sector – caring for the family alongside studying and/or a long commute to the university, were described as demanding. In addition to a high level of time and organisational commitment, relieving factors were also mentioned when, for example, it became clear in clinical work what the theoretical content of the course was useful for in practice.

#### 3.1.3. Stressors in clinical work

As medical students also gain clinical experience (e.g. nursing internships) at an early stage, and since the majority of the students surveyed in this study had completed vocational training in the healthcare sector and worked in a clinical capacity on a part-time basis, *stressors in clinical work* were also addressed in the interviews. The following subcategories were identified: *work organisation, complexity of clinical tasks, interpersonal interaction, discrimination and the role expectations of being a doctor* (see figure 4 (Fig. 4)).

With regard to *work organisation*, health policy requirements; work intensification, particularly due to staff shortages; disruptions to work processes; excessive bureaucracy; cost pressure; and the associated limited scope for action were mentioned.

Perceived pressure to perform and responsibility, high demands on one’s own concentration and emotionally stressful patient cases are summarised in the *complexity of clinical tasks category*.

The interviewees described shortcomings in *interpersonal interactions* as stress-inducing, such as in interactions with superiors, within a team or with patients. Observations in this regard – without any personal involvement – were also described as stressful.

Half of the interviewees discussed observations and experiences of discrimination in the form of sexism, racism or bullying. The interviewees also described the lack of their own strategies for dealing with discrimination as stressful. Silent approval of behaviour contrary to their own values and of perceived power structures was mentioned. The topic of racism was presented in a very reflective way in the interviews. On the one hand, discrimination against “imperfect-German-speaking” colleagues was mentioned, while on the other hand, attention was drawn to the dangers that can arise due to a lack of language skills.

Also discussed was the high *role expectations* of flawless and functioning doctors, which created additional pressure among students.

### 3.2. Individual stress amplifiers

The individual stress amplifiers found in the interviews included *perfectionism, striving for independence, perseverance*, the *desire for recognition, a desire for security and control* and, in some cases, pre-existing psychological *vulnerability* (see figure 5 (Fig. 5)).

The most frequently mentioned factor was personal *perfectionism*. Almost all the respondents described their own sense of entitlement as very high and their experiences of failure as rather unknown. The pursuit of *independence* and the associated negation of the need for help were described, as well as *perseverance* as a maxim or the neglect of physiological needs, such as sufficient sleep. The *desire for recognition*, such as from parents, and the *desire for security and control* were described as further stress intensifiers. The latter was associated by the interviewees with the desire to be able to learn all the material. There appeared to be a special situation for interviewees, which was a very long wait for a university place. After many semesters of waiting, doubts or problems with the degree programme are then perceived as causing stress. Some interviewees also stated that they had already experienced a previous mental illness or episode but had overcome this with therapeutic help.

### 3.3. Stress reactions

Respondents named stress reactions in the following categories: *emotional, cognitive, behavioural, physical* and *social* (see figure 6 (Fig. 6)).

Both the students and junior doctors surveyed reported fears of exams and of failure, as well as panic attacks, loss of control, helplessness, despair and mental breakdowns (*emotional stress reactions*). *Cognitive stress reactions* included poor concentration, rumination, procrastination and a general feeling of being controlled by others. The following were classified as *behavioural stress reactions*: uncontrolled crying, “explosions” or irritability at maximum stress. These behavioural changes, which are perceptible to those around them, were described as particularly challenging for private relationships. For some interviewees, the feeling of having to function went hand in hand with disillusionment and their own increasing harshness towards others – even close confidants – with supposedly less stress.

*Physical stress reactions* included palpitations, trembling, dizziness and nausea as well as tension. These reactions occurred not only during acute stress situations but also as a state of exhaustion over several weeks, such as after-semester examinations. Interviewees also reported too little space for recovery phases due to studying for a follow-up examination, internships or a clinical traineeship or compensating for time spent working part-time. More than half of the interviewees described reduced quantity and quality of sleep as a physical stress reaction.

*Social stress reactions* were mentioned in the form of withdrawal and fewer resources for the needs of others in the private environment.

### 3.4. Coping strategies

The openly coded and inductively derived coping strategies could be categorised into the three strategies of *instrumental, mental *and *regenerative* in Kaluza’s stress management [11].

Optimised time and self-management, the formation of learning groups, working with various learning platforms and adapted learning strategies were named instrumental strategies during studies. However, some of these were only reflected upon in the short term and were only effective for certain subjects or situations.

*Mental strategies*, such as reviewing previous expectations, recognising one’s own limits and values, accepting circumstances and being grateful for the opportunity to study medicine were used if doing so was perceived as meaningful. In some cases, the interviewees described a resigned acceptance of some systemic circumstances as counterproductive to the application of mental strategies. This applied, for example, to dealing with discriminatory harassment in the workplace when verbal reactions with reference to one’s own boundaries are refrained from within clinical and hierarchical structures and in order not to jeopardise one’s own career.

However, successful cognitive re-evaluations were also described, such as with regard to the learning effects of a repeat examination or pride in the study place as a mental strategy.

*Regenerative strategies*, such as rewards, regular sport or playing music, played a major role. The question of how hobbies could be maintained concerned some of the students. Short, solitary or social relaxation and recuperation periods helped the interviewees, although for some interviewees, these were accompanied by a guilty conscience. During the examination and preparation period, almost all of the interviewees suspended these activities due to the need for regenerative breaks, even or especially during this time, contrary to their own reflections.

In addition, the *social support* category was supplemented in line with the data found (see figure 7 (Fig. 7)). Many interviewees mentioned personal conversations as a proven coping strategy. Depending on the occasion and the progress of their studies, family, good friends or partners were mentioned as private dialogue partners. The focus here was on “getting rid of” or “sorting out” what was stressful. Later in the degree programme, fellow students or colleagues were added. Their better identification with study- and clinic-related experiences was described as beneficial. Institutional and professional offers of help, such as approaching mentors, collegial support offers or psychological counselling centres – if available – were considered helpful for crises associated with an inability to act and were used by some respondents.

Dealing with stress reactions was perceived as difficult at the beginning of the degree programme, while as the degree programme progressed, various stress reactions were increasingly accepted as a normal part of medical studies and later professional life. On the one hand, this can be seen as a healthy mechanism in the context of professionalisation. On the other hand, the loss of empathy/compassion, for example, is considered a sign of stress in the development of burnout [21].

## 4. Discussion

Along the levels of stress according to Kaluza, a variety of stressors were identified in the study programme, in private life and in the clinic. 

The main s*tressors in studying* included the amount of material, perceived pressure to perform and take exams as well as semester exams and qualification milestones. This is in line with national and international findings [14], [15], [16], [22], so that for years, a reflective look at the amount of learning material, the complexity and the depth of teaching in medical curricula has been called for in the sense of relationship prevention [23], [24]. Slavin described in 2014 [25] that existing institutional programmes based on mindfulness training and strengthening regenerative resources alone are not sufficient and are often not anchored in curricula. Slavin (2014) therefore recommended curriculum adjustments. Positive effects in terms of improved satisfaction [22], such as a change in the assessment system away from numerical grading towards passing/failing, have been recommended several times as stress-reducing measures, but they have hardly been implemented to date [26], [27].

The extent to which new examination formats and assessments change how stress is experienced or, in the best case, reduce it remains unclear. In terms of behavioural prevention, instrumental strategies (e.g. forming learning groups) – which were mentioned by the interviewees as means to cope with stressors during their studies – can be supported by more explicit, low-threshold awareness-raising measures promoted by the faculty (e.g. as part of special courses on learning strategies).

The interview results show that stressors during studies are characterised by *private stressors* re flanked, such as transition problems or secondary employment, as described, for example, by Bergmann in 2019 [8]. According to our research, the influence of stressors during studies by stressors in other areas of life and vice versa has so far received little attention for medical students. Regarding the clinical environment, St. Pierre and Hofinger [13] described how private problems or a lack of rest as chronic stressors in an acute medical stress situation can impair the ability to act as a doctor. These interactions also appear to be transferable to medical studies. Against the backdrop of the rather stable socio-structural backgrounds (e.g. socioeconomic, educational or migration backgrounds) of medical students nationally [28] and internationally [23], [29], the healthcare of an increasingly diverse society is a challenge [30]. What has changed is the growing proportion of female medical students (68.8% in Germany in 2022 [31]). Regardless of gender, the improved compatibility of studies (as well as later clinical work) not only with the family but also with the professional sideline activities of non-privileged students can be achieved with approaches of “flexible medical education” [24], [32], such as formally regulated part-time study programmes in a hybrid format at a faster or slower pace. Flexible education has so far been used primarily at US and Asian universities – also at Goethe University Frankfurt [24] for individualised part-time medical studies – not only for parents but also for professional athletes, students with disabilities and carers. Regular and structured exchanges with mentors who share their experiences of studying medicine, act as positive and reflective role models for the identity formation of future doctors and can address topics such as the compatibility of studies and a career with other areas of life would also seem to make sense [33].

Identified stressors in clinical work, such as unfavourable experiences with medical role models, one’s own health or demarcation behaviour, serious incidents and dealing with sexual harassment, can be addressed in joint cooperation between faculty development and occupational health management at clinics. In 2021, for example, a psycho-social support (PSU) peer network was established at Augsburg University Hospital as a low-threshold collegial support service for acutely stressed hospital employees of all professions [34]. In 2022, a pilot project, Peer Support in Medical Education – a measure whose effectiveness has not yet been tested – was implemented in cooperation between *PSU akut e. V.* and the Augsburg Faculty of Medicine to provide psychosocial support for fellow students in stressful situations [35]. The literature [36] emphasises the potential of an institutional social support structure. The qualitative findings of the current study supported this insofar as social support – initially from family and friends but later increasingly from fellow students and colleagues – was perceived by the interviewees as a valuable social coping strategy for dealing with external stressors.

All of the individual stress intensifiers described by Kaluza [11] were found in the interview data. Here, the results are in line with those of Slavin [25] specifically for medical students, such as maladaptive perfectionism, negating one’s own needs or self-reproach in the event of perceived inadequate performance. In the sense of strengthening satisfaction with oneself [37], events firmly anchored in the curriculum for active reflection on one’s own learning and stress-enhancing behaviour can provide a basis for supporting students, particularly in the acquisition of mental coping strategies. Reflecting on stress-increasing attitudes and thought patterns and the possibility of transforming them into stress-reducing, favourable attitudes appear to be a central prerequisite for a possible change in behaviour in this regard [38]. *Regenerative strategies* (e.g. compensation through sport) were mentioned by the interviewees as important, especially in phases of exam preparation, but were also described as challenging to implement. In many places, university-wide activities – also for the promotion of mental health – are offered as part of university health management. The stronger ways of addressing *mental coping strategies* described above can help students reduce their tendency to self-optimise and continue using *regenerative coping strategies* without a guilty conscience. The need for a generalised coping strategy, which was also mentioned by the interviewees, is contradicted by the concept of stress management, according to Kaluza [39]. Here, students need a broad and flexible repertoire of strategies adapted to triggering stressors, which must be promoted, learned and continuously practised in the context of studying [39].

## 5. Critical reflection

The results were taken from a non-representative sample of 22 medical students, students in their practical year and junior doctors. The participants were aware of the focus of the study (experiencing stress during their studies); thus, people with increased feelings of stress and presumably less sustainable coping strategies may have felt particularly encouraged to participate. This was shown, among other things, by the fact that some interviewees stated that they had already undergone therapeutic treatment. Due to the cross-sectional design, it is also not possible to say whether the interviewees’ statements were snapshot statements or manifest perceptions. The recruitment of students in their practical year and junior doctors via private and collegial contacts may also have impaired the open discussion and complete expression of thoughts. The digital interviews with these participants may also have led to a greater distance than with people interviewed in person.

At the same time, extensive qualitative results were found that were saturated with content for the group, which may have sharpened the focus on students experiencing higher levels of stress. No other data on the mental health of the interviewees were used for the study. However, other data collected at the Augsburg site showed that around half of students start their studies in good mental health and that their mental health is maintained – at least in the first semester [40].

The stressors related to the organisation of the degree programme mentioned by the Augsburg students, such as the lack of old exams, may have been due to the fact that the model degree programme was still young and had not yet been fully established, but this can also be assumed for other locations or faculties going through curricular reforms.

## 6. Conclusion

The present study provides insights into the stressors, stress reactions and coping strategies experienced in medical studies and underpins qualitative findings described in the literature in this regard, and it can thus provide further indications for curricular adjustments, preventive faculty programmes and cooperation with, for example, occupational health management.

In terms of *behavioural prevention*, the discourse on a sensible reduction in workload, increased support in the transition phase at the beginning of a degree course and more flexible studying for medical students with families or working in a clinic must be continued. In terms of behavioural prevention, the ability to cope with stress from various causative areas must be consciously learned, continuously practised and reflected upon. For medical students, the consistent thematisation of mental strategies for dealing with repeatedly described stress intensifiers, such as their own perfectionism, appears relevant in this context.

It is known that many doctors and medical students do not accept the same professional support that they would recommend to their patients [41]. Recognising one’s own limits, regularly applying appropriate strategies and accepting help early on when needed seem to be challenging for students as well as for faculty members and clinics [42]. What is needed here is a greater awareness of the need to develop the skills required for adequate and sustainable coping strategies in the sense of practising self-care and responsibility, which would also benefit the quality of care provided to patients in the course of a medical career.

## Notes

### Authors’ ORCIDs

- Iris Warnken: [0009-0000-8497-2541]
- Sabine Polujanski: [0000-0003-1864-9505]
- Thomas Rotthoff: [0000-0002-5171-5941]
- Ann-Kathrin Schindler: [0000-0002-2293-2357]

### Funding

This work was funded by the Volkswagen Foundation as part of the third-party funded project “Depressiveness and Burnout among Physicians as a Risk for Health Care – Analysing and Promoting Self-Regulation and Well-Being of Medical Students as Preventive Factors” (AZ. 98539).

## Acknowledgements

The team of authors would like to thank all of the participating interviewees for their willingness to give us insights into their experiences of stress. We would also like to thank Patrizia Ungar for conducting the interviews and Melissa Özsoy for the secondary coding and discursive development of the category system.

## Competing interests

The authors declare that they have no competing interests. 

## Supplementary Material

Set of questions from the interview guide

## Figures and Tables

**Table 1:**
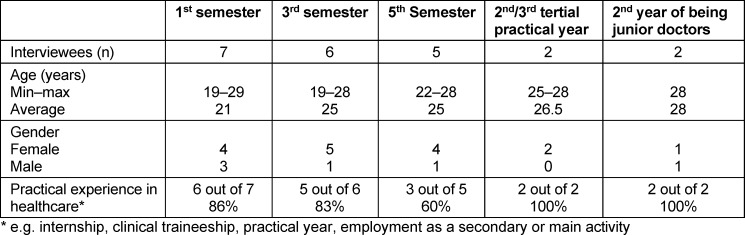
Sample description
