# Supplementary material for: Stress experience and coping strategies in medical studies – insights and a discussion of preventive measures
Source: GMS J Med Educ. 2025 Feb 17;42(1):Doc6. doi: 10.3205/zma001730 (PMC12086247; doi:10.3205/zma001730)
Supplement: Set of questions from the interview guide [file JME-42-6-s-001.pdf]

Attachment 1: Set of questions from the interview guide

In the course of the interview, there were several deviations from this in order to address or specifically enquire about topics

Key interview questions

1. Students from 2nd/3rd/5th semester: Crisis-like exceptional situations and/or permanent/long-lasting stressful situations:

- Which such situations have you experienced or noticed in yourself or in fellow students since the start of your studies?

- How was/is the situation perceived and how was/is it noticed?

- What symptoms of stress did you experience during the situation?

- Restriction of ability to act during the situation?

- Was/is it possible to fall back on coping strategies during the situation? To what extent?

- Which coping strategies were/are used? And how successful?

- What consequences did/does the situation have on studies/relationships/health/leisure activities?

- Were/are offers used to cope with the situation and/or to strengthen one's own (psychosocial) well-being?

- Were/are other people able to cope with this/such a situation? If so, how?

- Anticipate which exceptional crisis situations/stressful situations will/could occur in the further course of your studies.

- Who should you turn to if such a situation arises again? (fellow student – mentor – university student counselling service – clearing consultation – etc.)

2. Students in their practical year and junior doctors: Retrospective and clinical perspective

1) Key questions as for group 1, but here with a focus on the retrospective view of the study programme and, above all, inclusion of the clinical aspect:

- Crisis-like exceptional situations and permanent/prolonged stressful situations in the clinic (practical year itself, clinical traineeships, during own medical activities, with colleagues...)

2) Especially for junior doctors:

- Question about current experience of crisis-like exceptional situations and/or permanent/prolonged stressful situations in the profession and subsequent reference to the degree programme.

Attachment 1 to Warnken I, Polujanski S, Rotthoff T, Schindler AK. *Stress experience and coping strategies in medical studies – insights and a discussion of preventive measures.* GMS J Med Educ. 2025;42(1):Doc6. DOI: 10.3205/zma001730
